# Overexpression of cancer stem cell marker Lgr5 in colorectal cancer patients and association with clinicopathological findings

**DOI:** 10.22088/cjim.10.4.411

**Published:** 2019

**Authors:** Ramin Shekarriz, Fatemeh Montazer, Reza Alizadeh-Navaei

**Affiliations:** 1 Department of Hematology and Oncology, Gastrointestinal Cancer Research Center, Mazandaran University of Medical Science, Sari, Iran; 2 Department of Pathology, Iran University of Medical Sciences, Tehran, Iran; 3 Gastrointestinal Cancer Research Center, Mazandaran University of Medical Science, Sari, Iran

**Keywords:** Cancer stem cell, Lgr5, Colorectal cancer

## Abstract

**Background:**

To determine the expression of cancer stem cell marker Leucine-rich repeat-containing G-protein coupled receptor 5 (Lgr5) in colorectal carcinoma samples compared to normal adjacent tissue and any possible association with clinicopathological findings.

**Methods:**

This study was performed on forty samples of cancerous colorectal tissues (case group) and their adjacent normal mucosa (control group) in Imam Khomeini Hospital (Sari, Mazandaran, Iran). Expression of Lgr5 in tissue sections was done by immunohistochemistry. Statistical analysis was carried out using SPSS software.

**Results:**

Forty colorectal cancer patients including 21 males (57.8±11.6 years) and 19 females (58.4±12.77 years) were enrolled. Lgr5 was overexpressed in tumoral samples than normal adjacent tissues (77.5% vs 27.5%, p<0.001). Also, no association was found between primary tumor, regional lymph nodes, invasion, histological type, grade, distant metastasis and IHC results. Patients with low Lgr5 expression had a better survival rate than patients with high expression but this was not statistically significant (p=0.121).

**Conclusion:**

The higher immunoreactivity of Lgr5 in colorectal cancer tissues may indicate its role as a cancer stem cell marker in tumor carcinogenesis and patient’s survival however; Lgr5 is not associated with pathological prognostic variables.

Cancer has become a major health problem worldwide and along with obesity has been considered as a 21th-century epidemic (1). Colorectal cancer (CRC) is one of the commonest malignancies which ranks the third diagnosed cancer and the fourth cause of cancer related mortalities (2). CRC is noteworthy in several aspects including economic impact and financial burden on the healthcare system and a remarkable increase in mortality and incidence especially in young adults (3, 4). The emergence of treatment resistant cells, which is attributed to stem cells in tumor, is a critical barrier in cancer treatment that can eventually lead to failure in chemo-radiation therapy, recurrence and metastasis. Cancer stem cells (CSCs) were first described by Hope KJ et al (1977) in acute myeloid leukemia cell population Indeed, it is believed that cancerous tissue comprises of three cellular population similar to normal tissue: stem cells, proliferating and mature cells (5). These small subpopulations of tumoral cells share multiple features with normal stem cells including self-renewal and multi-potency abilities and are responsible for tumor initiation and transplantation, distant metastasis, tumor recurrence and drug resistance (6-8). Leucine-rich repeat-containing G-protein coupled receptor 5 (Lgr5) also known as GPR49 or GPR67 is a member of G protein-coupled receptors (GPCRs), highly important transmembrane proteins which are involved in cellular signal transduction. 

This multi-pass membrane protein is encoded by Lgr5 gene located on 12q21.1. Lgr5 consists of an extracellular N-terminal domain (containing leucine-rich repeats), transmembrane helices which pass membrane seven times and an intracellular N-terminal domain (9). Intestinal epithelium, to maintain integrity and homeostasis, is known to have a high regeneration rate which is guaranteed by the stem cells residing in the base of crypts (10). The accumulation of mutations in intestinal stem cells, the most notable in a tumor suppressor gene named adenomatous polyposis coli (APC), leads to over-expansion and proliferation of cells and is a key stage in the development of the pre-cancerous and tumoral lesions of the colon and rectum. Mutated APC gene results in over-activation of a signaling pathway named Wnt/β-catenin (11). Wnt/β-catenin is involved in different cellular functions including proliferation and migration accordingly is frequently dysregulated in cancers (12-14). In physiologic conditions Lgr5 acts to maintain intestinal homeostasis and cellular adhesion (15, 16). Also over-expression of Lgr5 will enhance Wnt/β-catenin (9, 17). Recently, researches have introduced Lgr5 as a potential adult cancer stem cell marker. Its oncogenic characteristics have been reported in various malignant conditions including cancers of cervix, breast, gastric, and colorectal which is responsible for cell proliferation, movement, invasion, drug resistance, and metastasis. Thus this study examined Lgr5 expression on CRC samples and any association with clinicopathological characteristics of tumor.

## Method

**Patients: **Paraffin-embedded tissues obtained from colectomy were selected from 40 colorectal cancer patients who referred to Imam Khomeini Hospital Sari, Iran. Pathological features including tumor type, histological grade, stage, perineural and vascular invasion, TNM categories of AJCC/UICC staging system and demographic data were extracted. We used normal mucosa adjacent to the tumor as control. Subjects had no prior history of chemotherapy or radiotherapy. The study contents were approved by the hospital ethics committee.

**LGR5 Immunohistochemistry staining protocol: **For immunohistochemical evaluations, formalin-fixed paraffin-embedded (FFPE) tissue blocks extracted from tumor lesions were used. Staining was performed on 5 μm thick sections mounted on slides organosilane-coated. The slides were washed twice in xylene (to eliminate paraffin), then were immersed in pure alcohol three times, washed with running water and immediately subjected to distilled water. Antigen retrieval was performed using citrate (30 min at 99° C, PH 6). After returning to the room temperature, slides were immersed for 10 minutes in 3% hydrogen peroxide. After returning to room temperature, slides were immersed in hydrogen peroxide 3% for 10 minutes. Afterwards they were incubated with rabbit polyclonal antibody to LGR5 (Biorbyt, Cambridge, UK) at 1: 200 and then washed with buffered saline solution. The next incubation was performed with a DAB-based chromogen solution for ten minutes in dark and samples were washed in distilled water. Positive immunohistochemical staining includes cases where the cells exhibited a brownish cytoplasm, irrespective of intensely. Five random fields at 400X magnification were determined for each sample. The scoring point was calculated as mentioned by Hou et al. (18) using sum of proportion score (0-25%=1, 26-50%=2, 51-75%=3, 76-100%=4) and intensity score (negative=0, mild=1+, moderate=2+ and strong=3+). This yielded a score ranging 1-7. The median expression score in tumor and adjacent samples was used to describe cut-off point. Therefore the scores ≥4 (median) were considered high expression and expression scores below 4 were categorized in low IHC group. 

Statistical analyses were performed with SPSS software (Version 18). Chi-square and Fisher's exact test were applied for qualitative data and t-test was used to analyze quantitative data. Life table method was applied to calculate survival and survival Kaplan-Meier method and log rank test was used to compare survival in high and low Lgr5 expression group. Results were considered as statistically significant if p-value was less than 0.05. 

## Results

Table 1 describes patient’s demographic and main clinical and pathological information according to their gender. As shown, there were no significant differences in the data of patients based on their gender. Each tumoral sample was examined by immunohistochemistry staining for Lgr5 expression compared to normal adjacent tissue. Cytoplasmic expression of Lgr5 was detected in all samples (including tumoral and non-tumoral). Expression levels based on the pathological findings are summarized in table 2. 

**Table 1:** Clinicopathological findings based on patient's gender

**Gender ** **Variable**	**Male**	**Female**	**P-value**
Age, year (mean ±SD)	57.8±11.6	58.4±12.77	0.87
*Tumor grade, n (%)*			
Well-differentiated Moderately differentiated poorly differentiated	18 (85.7%) 1 (4.8%) 2 (9.5%)	18 (94.7%) 1 (5.3%) 0 (0%)	0.73
*Histological subtypes, n (%)*			
Adenocarcinoma (conventional type) mucinous carcinoma	19 (90.5%) 2 (9.5%)	16 (84.2%) 3 (15.8%)	0.65
*Stage, n (%)*			
I II III IV	1 (4.8%) 10 (47.6) 3 (14.3%) 4 (19%)	3 (15.8%) 8 (42.1%) 4 (21%) 2 (10.5%)	0.64
*T, n (%)*			
T1, T2 T3	1 (4.8%) 20 (95.2%)	4 (21%) 15 (79%)	0.17
*N, n (%)*			
Nx N0 N1 N2	3 (14.3%) 13 (61.9%) 4 (19%) 1 (4.8%)	2 (10.5%) 11 (58%) 4 (21%) 2 (10.5%)	0.96
Distant metastasis*, n (%)* (positive)	4 (19%)	2 (10.5% )	0.66

**Table 2:** Lgr5 expression in relation to clinicopathological data

**Variable **	**High Lgr5 expression** **Sum of IHC score ≥4)** **)**	**Low Lgr5 expression ** **Sum of IHC score<4)** **)**	**P-value**
Samples n (%)			
CRC Normal adjacent tissue	31 (73.8%) 11 (26.2%)	9 (23.7%) 29 (76.3%)	P< 0.001
Patient, n (%)			
Male Female	17 (54.8%) 14 (45.2%)	4 (44.5%) 5 (55.5%)	0.71
Tumor grade, n (%)			
Well-differentiated Moderately differentiated Poorly differentiated	28 (90.3%) 2 (6.5%) 1 (3.2%)	8 (88.9%) 0 (0%) 1 (11.1%)	0.66
Perineural and vascular invasion,* n (%)*	4 (12.9%)	1 (11.1%)	1
N, n (%)			
N0 N1,N2	19 (61.3%) 8 (25.8%)	5 (55.5%) 3 (33.5%)	0.68
T, n (%)			
T1, T2 T3	4 (12.9%) 27 (87.1%)	1 (11.1%) 8 (88.9%)	1
Stage, n (%)			
I II III IV	3 (9.7%) 14 (45.16%) 4 (12.9%) 6 (19.35%)	1 (11.11) 4 (44.5%) 3 (33.3%) 0 (0%)	0.37
Distant metastasis (positive),* n (%)*	6 (19.35%)	0 (0%)	0.30

**Figure 1:**
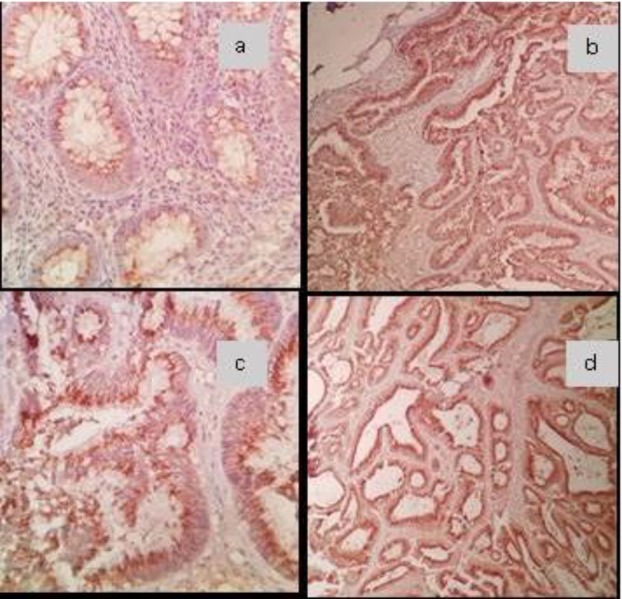
IHC study, (a) weak to moderately cytoplasmic Lgr5 staining in normal colon tissue (x400), (b,c) moderately cytoplasmic staining in colon cancer cells ×100 and x400 respectively; (d) strongly cytoplasmic staining in colon cancer cells (x100).

**Figure 2:**
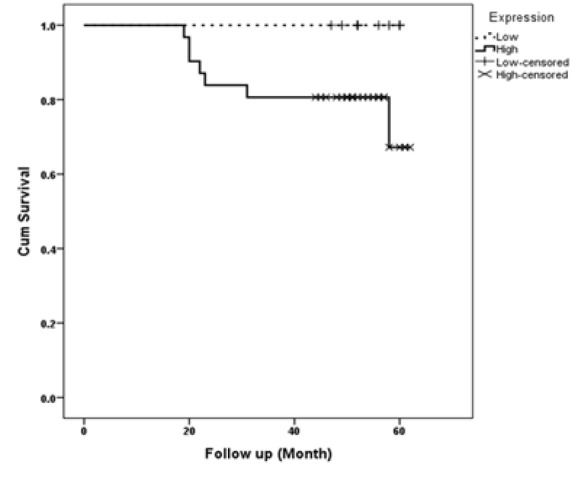
Kaplan-Meier curve for low and high expression of Lgr5 in colorectal cancer patients (P=0.121)

Higher expression of Lgr5 was observed in tumoral samples when compared to normal adjacent tissues (P<0.001). There were no other statistically differences between expression categories and clinicopathological findings. In figure 1, the expression patterns of Lgr5 in both tumoral and non-tumoral samples are shown. Seven patients died due to CRC and all of them were in high Lgr5 expression group (P=0.121). One, three and five year survival in high expression group was 100%, 81% and 75%, respectively. Survival examination of CRC patients based on Lgr5 expression is presented in figure 2.

## Discussion

The North of Iran has been reported as a region with high incidence of CRC. Although the incidence of this disease in Iran is low compared to Western countries, an increase in trend and incidence in younger Iranian people should be taken into consideration (19). It is worth mentioning that CRC is usually diagnosed at advanced stages with unfavorable prognosis in Iran (20) Therefore, identifying biomarkers that can be considered as potential therapeutic or diagnostic targets is of particular importance. We demonstrate a higher expression of Lgr5 in this tumor-normal comparison study. In cervical cancer cell lines, Cao et al showed that Lgr5 overexpression results in the tumorsphere formation, drug resistance to cisplatin and invasion (21). 

An IHC study by Hou demonstrated LGR5 and β-catenin expression in 126 breast cancer patients. 46% of samples revealed high Lgr5 expression which was associated with lymph node metastasis, size of the tumor and triple negative status. Also, the higher simultaneous expression of Lgr5 and β-catenin resulted in shorter relapse-free survival and poor prognosis (18). 

There is a similar report in colon cancer where both mice model and patients with Lgr5 positive tumors had decreased survival rates (22). However, the results are inconsistent regarding the oncogenic role of Lgr5 as in mice mammary stem cells, Lgr5 was not a necessary component for tumorigenesis (tumor initiation) and progression (23). It was reported that decreasing the expression level of Lgr5 via siRNAs, promotes clonogenic ability and tumorigenicity while its overexpression leads to an increase in cell-cell adhesion and lower motility and introduced Lgr5 as a wnt signaling pathway suppressor (24). Xiang-Shan Fan in an IHC study of 132 samples (comprising 12 normal colon mucosa, 18 adenomas and 102 colorectal cancer cases) showed a significantly higher expression of Lgr5 in cancerous tissues but similar to our observation, this expression was not associated with pathological characteristics like differentiation grade, TNM status, invasion or age of participants but a higher expression was determined in female subjects (25). 

In contrast vascular invasion, lymph node metastasis, and TNM stages were associated with elevated Lgr5 expression but no relation was found with CRC patient gender, age, tumor grade, and metastasis in the study conducted by He S et al. also the patients in low expression group had higher survival rate (26). In gastric cancer tissues, contrary to the findings mentioned above, Lgr5 expression was lower in samples from patients when compared to normal gastric mucosa. TNM stage, gender, and invasion were not influenced by Lgr5 expression (27). Resistance to chemotherapy regimen is a critical issue in oncology and may assist cancer relapse and metastasis (28). Various studies have been conducted on the role of cancer stem cells in developing drug resistance. In fact, the inherent ability of CSCs to repair the damaged genome and self-renewal properties are responsible for the development of resistance to chemo-radiotherapy (29). 

In this regard, Hsu et al. observed the significant association between high Lgr5 expression in CRC patients and unfavorable response to 5-fluorouracil based treatment. Overexpression of Lgr5 was not only associated with chemo-resistance but also with metastasis and cancer stage (30). A systematic review and meta-analysis of 7 studies (4 from China, 2 from Japan, 1 from the USA) concerning the relationship between lgr5 and CRC survival, reported the association between higher Lgr5 expression and lower overall survival (OS). This was observed for OS in Asian studies and not the USA (31). We found that patients with higher Lgr5 expression had shorter survival than that in low expression group, however, this trend *did not reach statistical significance* which may be due to sample size. 

We declare that the present study has some limitations that should be addressed. We used non-tumoral tissues adjacent to tumor as control specimens. Although immunochemical studies use adjacent tissues frequently, there is some evidence of molecular and transcriptome alterations in them which may differ normal tissues (32, 33). A relatively small sample size is another limitation. Finally, we found overexpression of Lgr5 in CRC tissues and its impact on survival but no association regarding sex and pathological data.
